# Angina Ludovici

**Published:** 1896-10-31

**Authors:** E. Percy Paton

**Affiliations:** Surgical Registrar to Westminster Hospital, &c.


### ANGINA LUDOVICI.

By E. Percy Paton, M.D., M.S., F.R.C.S., Surgical Registrar to Westminster Hospital, &c.

Angina Ludovici was so called because it was first fully described by Dr. Ludwig, of Stuttgart, in 1836, though previous to that date several cases had been described by James, of Exeter, and others. A better name for the disease is, perhaps, submaxillary cellulitis, as this gives a clear indication of its nature and situation, which its older name does not; and, indeed, there seems no sufficient reason why these cases, from a purely pathological point of view, should be separated from forms of cellulitis to be found elsewhere, as there is no essential difference in the cellular inflammation from that found elsewhere. But on account of the important and serious complications which may occur clinically, owing to the near proximity of important structures, it is of considerable practical advantage to keep these cases specially in mind.

Several different causes may give rise to inflammation of the cervical cellular tissue, such as wounds, suppuration in the glands, periostitis, &c. That form, however, to which the name Angina Ludovici is applied has been described as an idiopathic inflammation; but although, as the term idiopathic implies, no obvious cause can be assigned, still there is commonly present in the patient some channel by which the micro-organisms, which are no doubt responsible for the inflammation, can find an entrance, and, as carious teeth are very frequently present, this seems a most likely route for the germs to get in by. Cases also have been described as especially prevalent during epidemic outbreaks of other forms of spreading inflammations, such as erysipelas, but of this there does not seem to be any clear evidence.

The clinical course of the disease is as follows: A patient, who may have been in a robust state of health, but more commonly has been somewhat out of health for some time, is attacked with malaise and general febrile symptoms, with most probably a slight soreness of the throat; within a short time—it may be almost at once, or within one or two days—the neck is noticed to be swollen. The swelling is situated in the middle line between the hyoid bone and lower jaw, and is of a hard, brawny nature; indeed, it is sometimes so hard that no pitting can be produced even by firm pressure. As it increases the brawniness spreads with usually fairly-defined limits upwards to the floor of the mouth (if that, indeed, be not one of the first places for it to be noticed), downwards towards the larynx, in which direction it may extend until the sternum be reached, and laterally towards the anterior border of the sterno-mastoid muscles; while, at the same time, the constitutional depression considerably increases, the temperature at the same time is raised, though not much above 102 deg. Fahr. as a rule, the inflammation being asthenic in type. At this stage, in mild cases, either by simple treatment, or without any special treatment, resolution may take place; or without much increase of the swelling it may soften and break down, and then a spontaneous cure follows.

Instead, however, of this favourable course, which occurs in the simpler cases, the tumefaction gradually still further increases, and, as it does so in an upward direction, the tongue is pushed up towards the hard palate, and at the same time considerably increases in size, owing probably to pressure on the veins by the brawny infiltration at its root, causing œdema, while in the neck the increase of swelling causes increased difficulty in opening the mouth and in swallowing.

The trouble having advanced thus far, the patient is now in a position of considerable danger of the onset of the most serious complication of this disease, namely, œdema of the glottis. The suddenness with which this may supervene is very great, so much so, that I myself have personal knowledge of a case in which the patient was admitted apparently not in any immediate risk, but died of asphyxia while being carried up the hospital stairs, before tracheotomy could be done, and records of many such cases might be quoted. Usually, however, there is warning of impending danger in the alteration of the voice and in the paroxysmal attacks of dyspnœa which may precede the fatal seizure which may follow if active treatment be not undertaken. Another serious complication is from the extensive spread of the inflammation until it reaches the mediastinum, setting up a fatal suppurative mediastinitis.

Two other complications must not be forgotten; they are septic infection and extensive sloughing. The latter may be very extensive, so that in the separation of the sloughs important structures may be laid bare, and in a patient already without any reserve of strength the strain of the slough separation and consequent suppuration may be too much for him, not to speak of the local injury should recovery occur. Of the septic dangers it is only needful to remark that they do not differ from those which are present when a similar source of infection exists from any other cause.

Having detailed above the dangers to which this affection may give rise, it is satisfactory to be able to turn to the consideration of the treatment by which, if it be undertaken promptly and efficiently carried out, these complications may be prevented, and the disease in most, if not in all cases, brought to a satisfactory termination.

In the mild cases, in which the swelling in the neck is only small and just commencing, it is not at once necessary to proceed to operative treatment, but such palliative measures as a saline purge with frequently changed hot boracic acid fomentations applied locally over the swellng may be tried, and the result watched. If under this simple treatment the trouble does not increase, but on the contrary subsides, all is well. If, however, the swelling continues to increase, and more especially should the slightest sign of extension towards the base of the tongue or larynx occur, such as pushing up of the tongue towards the palate, and particularly alteration in the character of the voice or slight dyspnœa, waiting in the hope of spontaneous subsidence is no longer justifiable, and a free incision into the brawny swelling must at once be made. It is generally best to give an anæsthetic, though it must be remembered that if any considerable dyspnœa be present this may be increased by reducing the sensibility of the laryngeal mucous membrane, and so diminishing the action of the abductors of the vocal cords, which keep the glottis as wide as possible in cases of œdema glottidis. The incision itself should be made exactly in the middle line and should be free, extending from a little below the symphysis of the jaw almost to the hyoid bone, and should be steadily carried down till the deep cervical fascia has been cut through, as the mischief lies underneath this resistant membrane; the knife may then be laid aside, and the opening deepened by a steel director passed backwards and upwards towards the root of the tongue, the track thus made being enlarged by dressing forceps or the forefinger. A very considerable depth is easily reached here and with perfect safety; merely the loose tissue between the two genio-hyo-glossus muscles being separated, and there being no vessels of any size interfered with, the bleeding is almost *nil.* Generally these measures will give exit to a small quantity of very foul-smelling pus, but sometimes no pus escapes, and instead a small quantity of serum-like fluid comes away, and the depths of the opening have a greyish sloughy appearance.

In any case a considerable sized drainage tube should be inserted, and a large boracic acid fomentation applied which should be changed every three or four hours. Should there be any bleeding at first a dry dressing may be put on till this has stopped, and then the fomentations may be commenced. By this plan of treatment it will be found that the swelling will in almost all cases at once commence to subside, and in a comparatively short time all danger will have gone. In some cases, however, as in cellulitis elsewhere, the inflammatory process does not at once stop, in which case it is necessary to follow the swelling, as would be done elsewhere, with free incisions, avoiding, however, injury to the large vessels, &c., in the neck, which is best done by keeping as far as possible to the middle line.

As with these cases there is always a considerable general depression, a lowering plan of general treatment is not admissible, but after a good purge plenty of milk, beef tea, or even fish and other light meats should be given, as this materially shortens the convalescence, though at first, due to the swelling of the tongue, and the consequent difficulty of swallowing, fluid food only can be taken. In a few cases, especially in hospital practice, medical aid may be called in so late that when first seen very considerable dyspnœa is already present; when this is the case it will probably be wiser to do a temporary tracheotomy, while at the same time freely incising the brawny thickening, than to merely rely on the latter, with the possibility of increased œdema of the larynx coming on suddenly when skilled aid is not immediately at hand.

